# Laparoscope combined with ureteroscope in the treatment of bile duct stones and cholecystolithiasis in a child: a case report and literature review

**DOI:** 10.3389/fmed.2024.1429127

**Published:** 2024-10-03

**Authors:** Chang Fu, Hengwei Jin, Lijun Fang, Hongji Xu

**Affiliations:** ^1^Department of Hepatobiliary and Pancreatic Surgery, General Surgery Center, The First Hospital of Jilin University, Changchun, China; ^2^Department of General Surgery, Liaoyuan People’s Hospital, Liaoyuan, China; ^3^Department of Abdominal Surgery, Guiqian International General Hospital, Guiyang, China

**Keywords:** cholecystolithiasis, bile duct stones, pediatric surgery, endoscopic retrograde cholangiopancreatography, case study, choledocholithiasis, gallstones

## Abstract

Cholecystolithiasis combined with bile duct stones is more and more common in children, but the surgical treatment is still controversial. We report on a 3-year-old boy, who underwent laparoscope combined with ureteroscope for choledocholithiasis with cholecystolithiasis. This combination therapy offers the possibility to treat pediatric patients with cholecystolithiasis and bile duct stones in low-resource settings where ERCP experience and child-specific surgical instruments are not available. Additionally, a comprehensive review of previous studies was conducted to summarize the surgical treatments. The surgical treatment of children should be made according to the specific situation to maximize the success of the operation and reduce the risk.

## Introduction

Cholecystolithiasis are one of the most common diseases in the digestive system, with a notable rise in incidence observed among pediatric patients in recent years (1–3). Bile duct stones (choledocholithiasis) are often secondary to cholecystolithiasis, often leading to severe symptoms such as abdominal pain, jaundice, and cholangitis. In the past, open surgery was the primary approach for treatment, albeit associated with prolonged surgical trauma and recovery periods, imposing significant burdens on both children and their families. Nowadays, with the development and combined application of laparoscopic and endoscopic techniques, minimally invasive treatment in children has achieved remarkable results. We reported a successful case of using flexible ureteroscope for bile duct exploration to treat bile duct stones. This case not only contributes valuable clinical experience but also underscores the advantages of employing multiple endoscopic techniques in tandem.

## Case presentation

**Figure 1:**
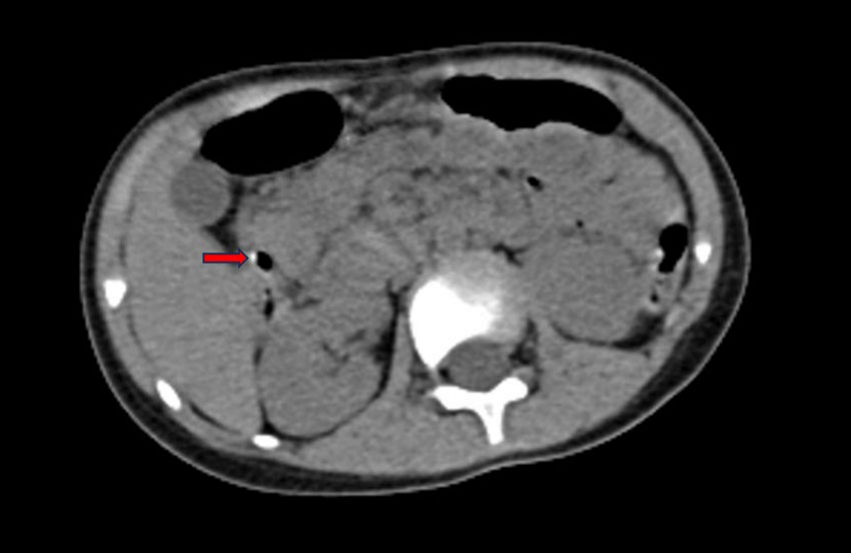
CT showed bile duct stones and dilated bile duct. The red arrow indicates clearly visible bile duct stones.

A 3-year-old boy was referred to our hospital due to persisting abdominal pain after 3 days of symptomatic treatment in local hospital. The patient’s past and family history were normal. Physical examination revealed no jaundice, but notable tenderness and rebound tenderness were observed in the right upper quadrant. Laboratory examination revealed normal levels of total bilirubin and direct bilirubin. However, *γ*-glutamyl transpeptidase was elevated at 160.2 U/L, while levels of aspartate aminotransferase and alanine aminotransferase were elevated at 66.8 U/L and 164.4 U/L, respectively. Alkaline phosphatase was also elevated at 317.9 U/L. The leukocyte count was 9.83×10^9^/L, and the C-reactive protein (CRP) level was 8 mg/L. Abdominal ultrasound showed that the bile duct stone was located in the pancreatic segment of the common bile duct (CBD), measuring approximately 8 mm, accompanied by a dilatation of the CBD of approximately 9 mm. Abdominal computed tomography (CT) showed cholecystolithiasis, bile duct stones and dilatation of intrahepatic and extrahepatic bile ducts (Figure 1). Magnetic resonance cholangiopancreatography (MRCP) was unsuccessful because the child was unable to cooperate. Considering the potential increased risk of developmental delays, attention deficit hyperactivity disorder (ADHD), and psychiatric disorders, we opted not to perform MRCP under general anesthesia (4).

**Figure 2:**
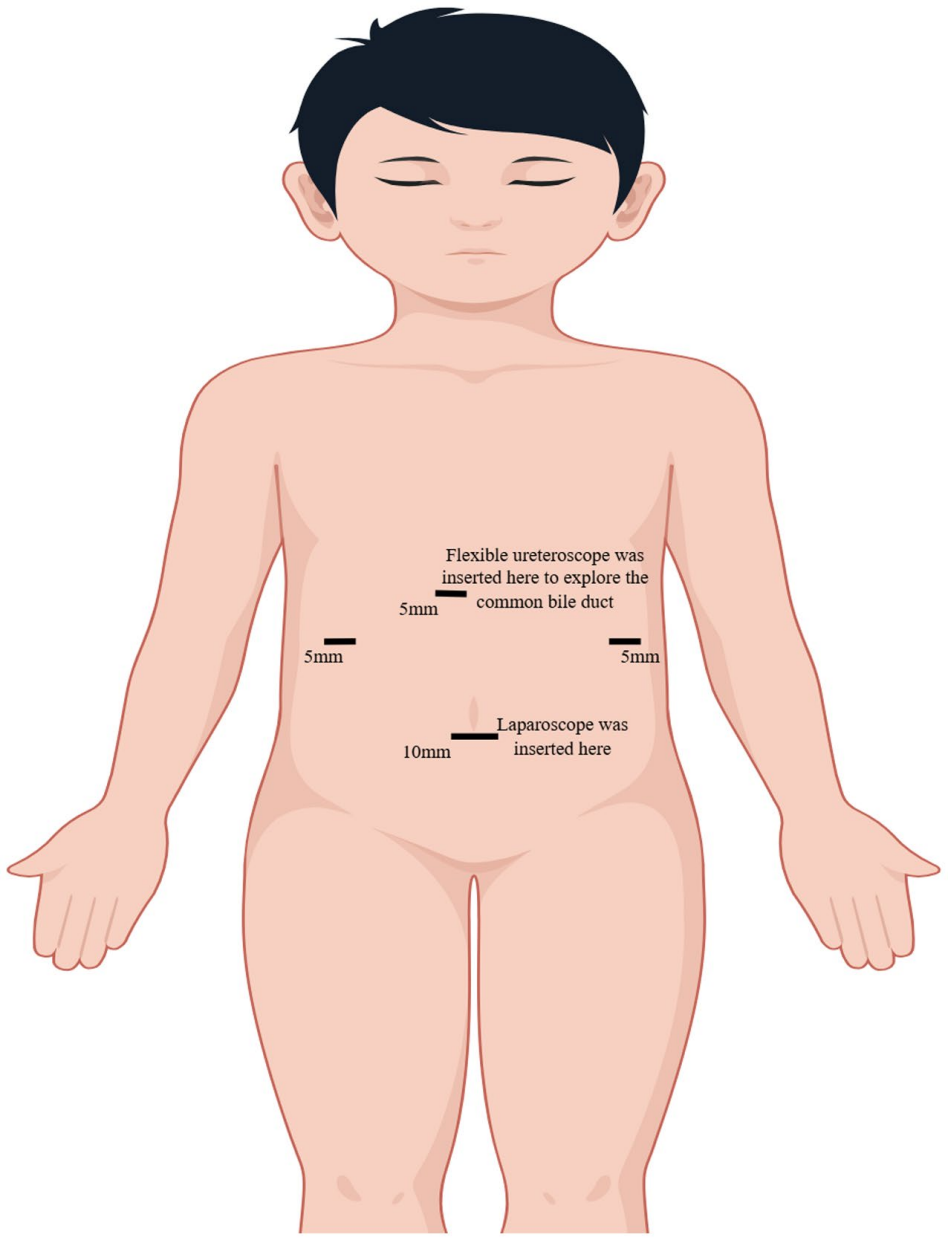
Port sites. By Figdraw.

**Figure 3:**
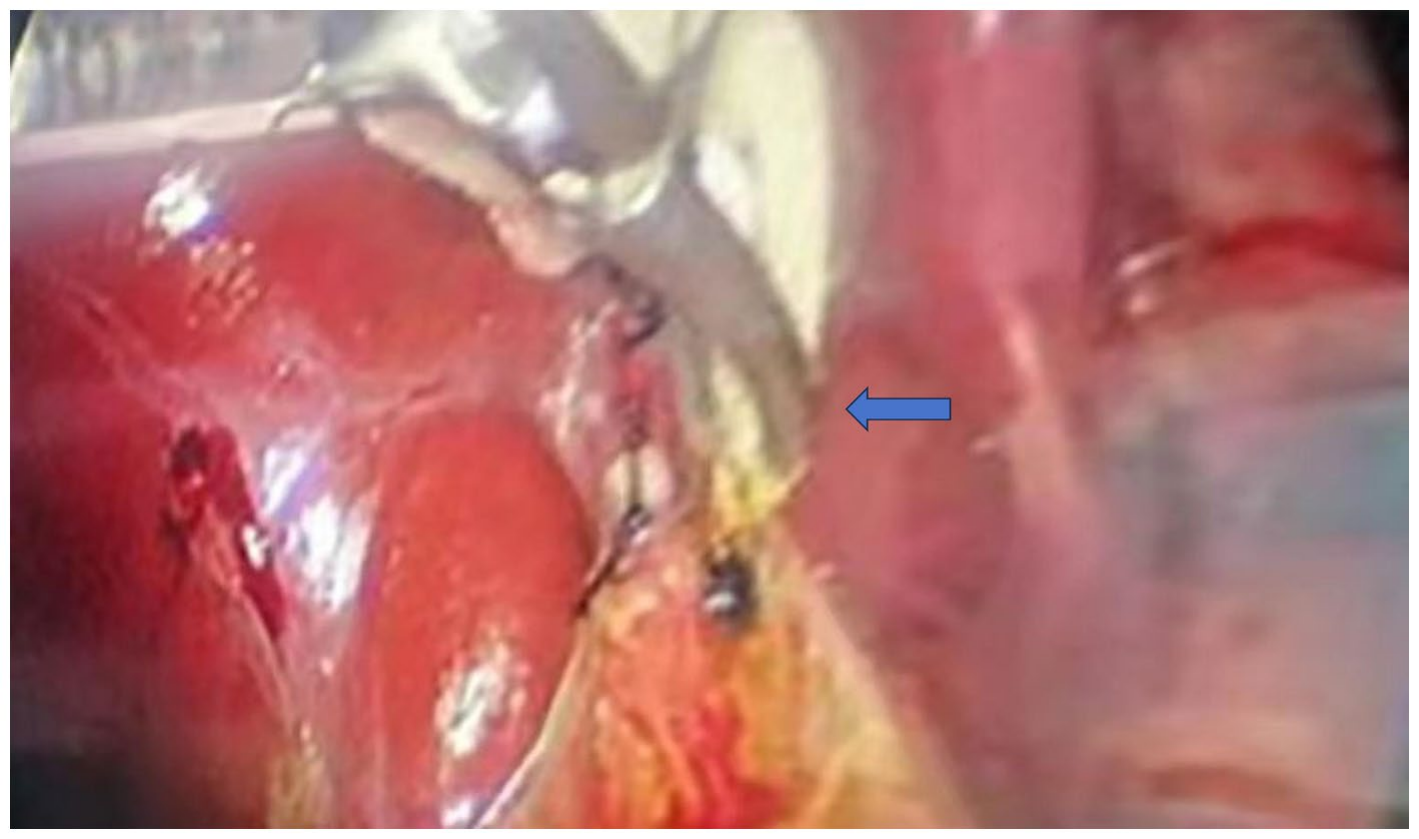
Flexible ureteroscope was inserted into the common bile duct under the guidance of grasping forceps during the operation. The blue arrow indicates the forceps guiding the flexible ureteroscope during bile duct exploration.

**Figure 4:**
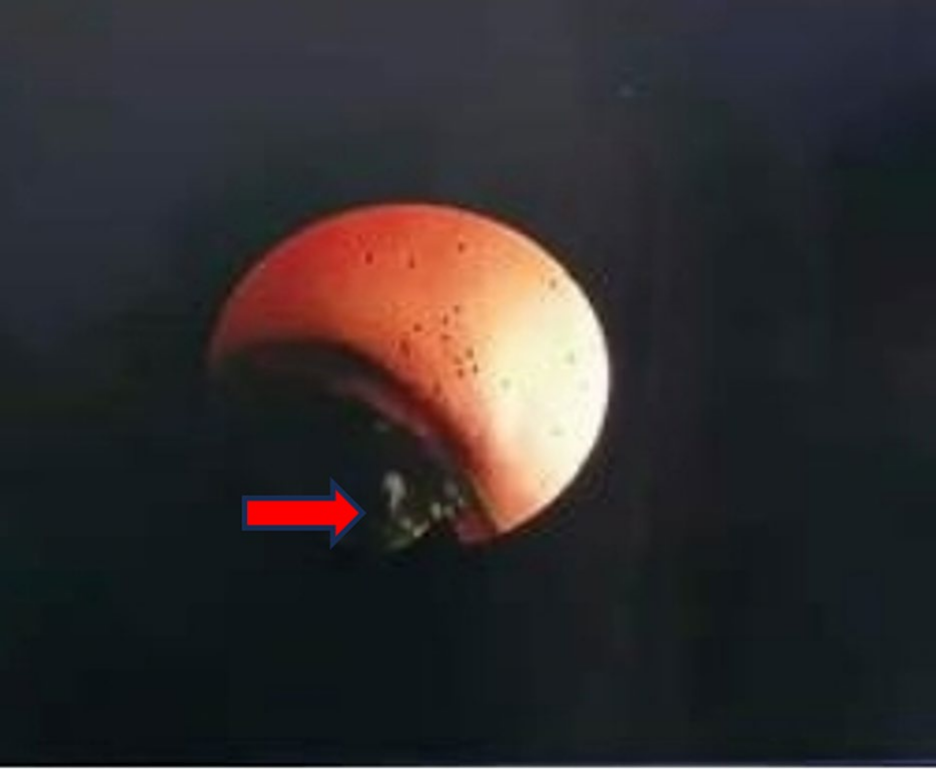
Bile duct exploration showed common bile duct stones. The red arrow points to the bile duct stones as seen in the ureteroscope’s field of view.

**Figure 5:**
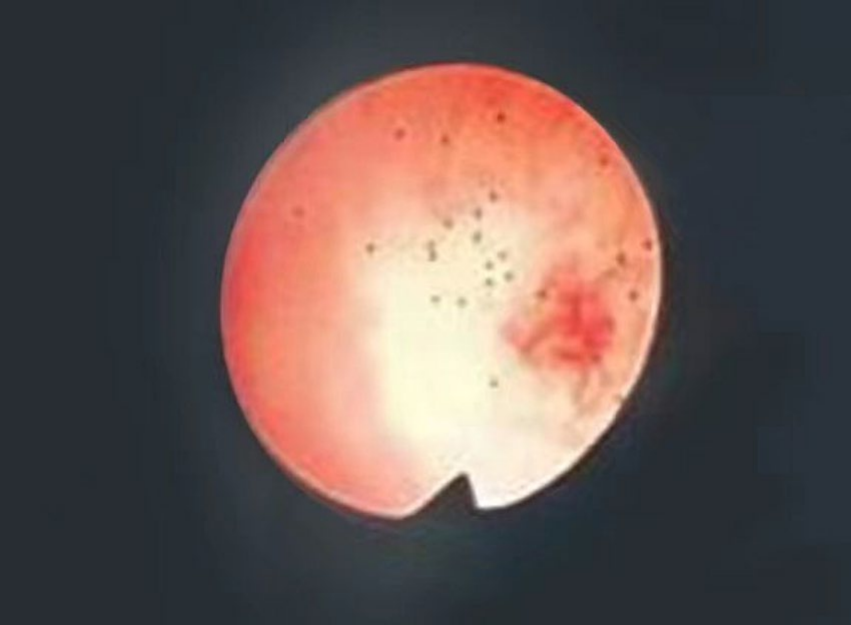
The bile duct stones were removed under ureteroscope. The ureteroscope’s field of view shows a clear bile duct with no residual stones.

Due to the pronounced symptoms of abdominal pain and alterations in blood routine and liver function, we opted for laparoscopic cholecystectomy (LC) in conjunction with bile duct exploration using a flexible ureteroscope. The patient received a preoperative intravenous injection of cefotaxime to prevent infection. A 10 mm camera port, fitted with a blunt trocar, was inserted above the umbilicus through a small incision. Three 5 mm ports were inserted under direct vision, one near the xiphoid, and the others in the right and the left flank (Figure 2). We exposed the infundibulum and cystic duct, utilizing a second forceps introduced through the right flank port to grasp the infundibulum and expose the triangle of Calot. Subsequently, we isolated the cystic duct, the CBD, and the cystic artery. After dissection of the cystic duct and artery, the cystic artery was clipped and transected. The CBD was longitudinally incised by 0.8 cm at its upper portion. Under direct laparoscopic visualization, a 6.5Fr flexible ureteroscope was inserted through the trocar hole beneath the xiphoid. A grasp forceps was used to guide the flexible ureteroscope into the CBD and slowly enter the scope for exploration (Figure 3). After identifying the stones, they were carefully extracted using a stone retrieval basket under the guidance of the ureteroscope (Figure 4). Following exploration, the flexible ureteroscope was retracted to ensure no residual stones remained in the CBD (Figure 5). During the operation, mild bile duct inflammation was observed and the stones were completely removed. Considering the risk of T-tube dislodgement in the pediatric patient, we performed simple interrupted everting suturing of the CBD using 5–0 PDS II sutures without placing a T-tube drain. The patient had an uneventful postoperative recovery and was discharged 4 days later. During the three-year follow-up period after surgery, the child did not experience a recurrence of bile duct stones.

## Discussion

The prevalence of gallstones in adults is approximately 10 to 15%, with 10 to 20% of these individuals also having bile duct stones (5, 6). Compared to adults, the incidence in children is lower, at approximately 1.9 to 4% (7). Potential factors contributing to cholelithiasis in children encompass obesity, hemolytic disease, premature birth, and reliance on total parenteral nutrition (8–10). Unlike adults, children with gallstones have more obvious symptoms (1, 11, 12). However, clinical presentations in pediatric cases are frequently nonspecific, compounded by limited expressiveness in children, thus increasing the likelihood of missed diagnosis or misdiagnosis (12). Ultrasonography is the preferred examination method. When bile duct dilatation exceeds 6 mm, there is a high probability of bile duct stones (13). For children, it is also important to consider differentiation from choledochal cysts and pancreaticobiliary maljunction. MRCP and cholangiography can enhance diagnostic sensitivity and specificity.

At present, there is no consensus on the optimal surgical method for treating cholecystolithiasis and bile duct stones in pediatrics. Therapeutic techniques include laparotomy, laparoscopic surgery, percutaneous intervention, and endoscopic retrograde cholangiopancreatography (ERCP) (14). With the advancement of endoscopic technology, the utilization of ERCP for bile duct stones treatment in children is progressively increasing (15–17). LC + ERCP are two independent surgical procedures, necessitating coordinated efforts among multiple teams and amplifying the anesthesia-related risks (18). Children undergoing ERCP treatment face radiation exposure, which may negatively impact their growth and development and increase the risk of cancer (19). Moreover, due to the fragile development of the duodenal papilla in children, surgical incisions and dilations may compromise its normal function. Additionally, ERCP poses potential serious complications such as pancreatitis and duodenal perforation (20). We also need to recognize that the situations in which ERCP is applicable are also limited by a lack of medical resources and operator’s expertise.

Recent study had shown that ERCP and laparoscopic common bile duct exploration (LCBDE) can be safely and effectively used in children (21). Laparoscopic bile duct exploration in children with bile duct stones encompasses various techniques, including combination with choledochoscope, cystoscope, and ureteroscope. In addition, Destro et al. also applied Virtual Reality combined with minimally invasive surgical techniques to the preoperative evaluation of pediatric patients, providing a new perspective for the diagnosis and treatment of pediatric hepatobiliary diseases (22). By leveraging the technical advantages of duodenoscopy, laparoscopy, choledochoscopy, or ureteroscope, the diagnosis and treatment of pediatric patients with bile duct stones can be tailored to achieve individualized and minimally invasive therapeutic goals. Compared with LC+ ERCP, this treatment can decrease length of stay and the frequency of ductal stent placement (23–26). In our study, laparoscopy and 6.5Fr thin flexible ureteroscope were used according to the condition of the child. The slender profile of the thin ureteroscope facilitates smoother insertion into the bile duct, reducing procedural difficulty. Moreover, its design ensures ample space between the lens body and the bile duct, thereby enhancing the success rate of both operation and stone removal while significantly mitigating the risk of bile duct injury. By utilizing flexible ureteroscope for choledoscopy, we avoided the potential injuries caused by direct clamping, thereby enhancing the safety and reliability of the procedure. Rothstein et al. also suggested that combined ureteroscope may be helpful depending on the patient’s body size (27).

To comprehensively investigate and consolidate the management of cholecystolithiasis combined with bile duct stones in children, we conducted a thorough review and analysis of prior studies (18, 25, 28–44) (Supplementary Table 1). Our findings indicate that a majority of pediatric patients typically present with abdominal pain, often accompanied by a history of inherited hematologic disorders. Laboratory investigations commonly reveal elevated levels of total bilirubin and liver enzymes. Furthermore, ultrasound or imaging examinations frequently detect bile duct dilatation and stones. The main surgical methods were LC + ERCP or LCBDE, but the specific approaches or surgical instruments were different. For example, choledochoscope, ureteroscope, cystoscope and so on can be used in bile duct exploration. Moreover, the operative approach may involve access via the cystic duct or the CBD. For LC + ERCP, the sequence of the two procedures also differed among studies. In addition, we also note that for these two surgical methods, we need to avoid the occurrence of complications such as bleeding, pancreatitis, and retained stones.

At present, there is no clinical guideline for the treatment of children with cholecystolithiasis and bile duct stones. This article aims to provide a feasible surgical treatment for children and summarize previous methods to enhance awareness among medical practitioners. The selection of specific clinical treatment should be individualized based on comprehensive consideration of the patient’s condition, cost-effectiveness, safety and feasibility of surgical plans, which is worthy of further exploration and practice.

## Data Availability

The raw data supporting the conclusions of this article will be made available by the authors on request.

## References

[1] Murphy PB Vogt KN Winick-Ng J McClure JA Welk B Jones SA. The increasing incidence of gallbladder disease in children: a 20year perspective. J Pediatr Surg. (2016) 51:748–52. doi: 10.1016/j.jpedsurg.2016.02.017, PMID: 26951963

[2] Walker SK Maki AC Cannon RM Foley DS Wilson KM Galganski LA. Etiology and incidence of pediatric gallbladder disease. Surgery. (2013) 154:927–33. doi: 10.1016/j.surg.2013.04.040 24074432

[3] Pogorelić Z Aralica M Jukić M Žitko V Despot R Jurić I. Gallbladder disease in children: a 20-year single-center experience. Indian Pediatr. (2019) 56:384–6. doi: 10.1007/s13312-019-1535-1, PMID: 30898989

[4] Ing C Sun M Olfson M DiMaggio CJ Sun LS Wall MM. Age at exposure to surgery and anesthesia in children and association with mental disorder diagnosis. Anesth Analg. (2017) 125:1988–98. doi: 10.1213/ANE.0000000000002423, PMID: 28857799 PMC5856466

[5] Stinton LM Shaffer EA. Epidemiology of gallbladder disease: cholelithiasis and cancer. Gut Liver. (2012) 6:172–87. doi: 10.5009/gnl.2012.6.2.172, PMID: 22570746 PMC3343155

[6] Tazuma S. Gallstone disease: epidemiology, pathogenesis, and classification of biliary stones (common bile duct and intrahepatic). Best Pract Res Clin Gastroenterol. (2006) 20:1075–83. doi: 10.1016/j.bpg.2006.05.009, PMID: 17127189

[7] Mehta S Lopez ME Chumpitazi BP Mazziotti MV Brandt ML Fishman DS. Clinical characteristics and risk factors for symptomatic pediatric gallbladder disease. Pediatrics. (2012) 129:e82–8. doi: 10.1542/peds.2011-0579, PMID: 22157135

[8] Fradin K Racine AD Belamarich PF. Obesity and symptomatic cholelithiasis in childhood: epidemiologic and case-control evidence for a strong relation. J Pediatr Gastroenterol Nutr. (2014) 58:102–6. doi: 10.1097/MPG.0b013e3182a939cf, PMID: 23969538

[9] Koebnick C Smith N Black MH Porter AH Richie BA Hudson S. Pediatric obesity and gallstone disease. J Pediatr Gastroenterol Nutr. (2012) 55:328–33. doi: 10.1097/MPG.0b013e31824d256f, PMID: 22314396 PMC3401629

[10] Zdanowicz K Daniluk J Lebensztejn DM Daniluk U. The etiology of Cholelithiasis in children and adolescents-a literature review. Int J Mol Sci. (2022) 23:13376. doi: 10.3390/ijms232113376, PMID: 36362164 PMC9657413

[11] Herzog D Bouchard G. High rate of complicated idiopathic gallstone disease in pediatric patients of a north American tertiary care center. World J Gastroenterol. (2008) 14:1544–8. doi: 10.3748/wjg.14.1544, PMID: 18330945 PMC2693749

[12] Bogue CO Murphy AJ Gerstle JT Moineddin R Daneman A. Risk factors, complications, and outcomes of gallstones in children: a single-center review. J Pediatr Gastroenterol Nutr. (2010) 50:303–8. doi: 10.1097/MPG.0b013e3181b99c72, PMID: 20118803

[13] Samara O Azzam MI Alshrouf MA Khanfar AN Mohialdeen RR. Diagnostic accuracy of ultrasonography compared with magnetic resonance cholangiopancreatography in the detection of choledocholithiasis. J Clin Ultrasound. (2022) 50:247–53. doi: 10.1002/jcu.23136, PMID: 34995366

[14] Kim H Shin SP Hwang JW Lee JW. Outcomes of laparoscopic common bile duct exploration (LCBDE) after failed endoscopic retrograde cholangiopancreatography versus primary LCBDE for managing cholecystocholedocholithiasis. J Int Med Res. (2020) 48:300060520957560. doi: 10.1177/0300060520957560, PMID: 33059506 PMC7580163

[15] Taj MA Leghari A Qureshi S Ghazanfar S Niaz SK Quraishy MS. Endoscopic retrograde cholangiopancreatography: a therapeutic modality in children and adolescents. J Pak Med Assoc. (2012) 62:98–101. PMID: 22755366

[16] Dişçi E Peksöz R Yıldız M Yıldırgan M Albayrak Y Fakirullahoğlu M. Endoscopic retrograde cholangiopancreatography in pediatric patients. J Laparoendosc Adv Surg Tech A. (2022) 32:320–4. doi: 10.1089/lap.2021.0517 35041496

[17] Mercier C Pioche M Albuisson E Ponchon T Gonzalez JM Barthet M. Safety of endoscopic retrograde cholangiopancreatography in the pediatric population: a multicenter study. Endoscopy. (2021) 53:586–94. doi: 10.1055/a-1209-0155, PMID: 32599632

[18] Hill SJ Wulkan ML Parker PM Jones TK Heiss KF Clifton MS. Management of the pediatric patient with choledocholithiasis in an era of advanced minimally invasive techniques. J Laparoendosc Adv Surg Tech A. (2014) 24:38–42. doi: 10.1089/lap.2013.0306, PMID: 24380575

[19] Baron TH Schueler BA. Pregnancy and radiation exposure during therapeutic ERCP: time to put the baby to bed? Gastrointest Endosc. (2009) 69:832–4. doi: 10.1016/j.gie.2008.07.010, PMID: 19327473

[20] Prasil P Laberge JM Barkun A Flageole H. Endoscopic retrograde cholangiopancreatography in children: a surgeon's perspective. J Pediatr Surg. (2001) 36:733–5. doi: 10.1053/jpsu.2001.22948 11329577

[21] Bosley ME Zamora IJ Neff LP. Choledocholithiasis-a new clinical pathway. Transl Gastroenterol Hepatol. (2021) 6:35. doi: 10.21037/tgh-20-172, PMID: 34423156 PMC8343507

[22] Destro F Salerno R Calcaterra V Ardizzone S Meroni M Roveri M. Echo-endoscopy combined with virtual reality: a whole perspective of laparoscopic common bile duct exploration in children. Child Aust. (2023) 10:760. doi: 10.3390/children10040760, PMID: 37190009 PMC10137240

[23] Bosley ME Gaffley MWG Zeller KA Sieren LM Petty JK Pranikoff T. Balloon sphincteroplasty in pediatric laparoscopic common bile duct exploration. J Pediatr Surg. (2021) 56:825–8. doi: 10.1016/j.jpedsurg.2020.12.001, PMID: 33349422

[24] Wandling MW Hungness ES Pavey ES Stulberg JJ Schwab B Yang AD. Nationwide assessment of trends in Choledocholithiasis Management in the United States from 1998 to 2013. JAMA Surg. (2016) 151:1125–30. doi: 10.1001/jamasurg.2016.2059, PMID: 27556900

[25] Short SS Frykman PK Nguyen N Liu Q Berel D Wang KS. Laparoscopic common bile duct exploration in children is associated with decreased cost and length of stay: results of a two-center analysis. J Pediatr Surg. (2013) 48:215–20. doi: 10.1016/j.jpedsurg.2012.10.041, PMID: 23331818

[26] Sardiwalla II Koto MZ Kumar N Balabyeki MA. Laparoscopic common bile duct exploration use of a rigid Ureteroscope: a single institute experience. J Laparoendosc Adv Surg Tech A. (2018) 28:1169–73. doi: 10.1089/lap.2018.0042, PMID: 29676951

[27] Rothstein DH Harmon CM. Gallbladder disease in children. Semin Pediatr Surg. (2016) 25:225–31. doi: 10.1053/j.sempedsurg.2016.05.005 27521713

[28] Rauh J Dantes G Wallace M Collings A Sanin GD Cambronero GE. Transcystic laparoscopic common bile duct exploration for pediatric patients with Choledocholithiasis: a multi-center study. J Pediatr Surg. (2024) 59:389–92. doi: 10.1016/j.jpedsurg.2023.10.046, PMID: 37957103

[29] Niknam R Mortazavi SMM Jahromi MG Davoodi M Soheili M Ataollahi M. Stone removal in a 5-year-old child with extrahepatic biliary obstruction using ERCP: a case report and a mini-review. Clin Case Reports. (2023) 11:e7620. doi: 10.1002/ccr3.7620, PMID: 37520769 PMC10374985

[30] Pogorelić Z Lovrić M Jukić M Perko Z. The laparoscopic cholecystectomy and common bile duct exploration: a single-step treatment of pediatric Cholelithiasis and Choledocholithiasis. Child Aust. (2022) 9:1583. doi: 10.3390/children9101583, PMID: 36291520 PMC9601212

[31] Cisarò F Pane A Scottoni F Pizzol A Romagnoli R Calvo PL. Laparo-endoscopic Rendez-Vous in the treatment of Cholecysto-Choledocolithiasis in the pediatric population. J Pediatr Gastroenterol Nutr. (2022) 74:819–22. doi: 10.1097/MPG.0000000000003444, PMID: 35258502

[32] Fishman DS Barth B Mazziotti MV Lazar DA Brandt ML Fallon SC. Same anesthesia endoscopic retrograde cholangiopancreatography and laparoscopic cholecystectomy: the pediatric ERCP database Intiative experience. J Pediatr Gastroenterol Nutr. (2020) 71:203–7. doi: 10.1097/MPG.0000000000002722, PMID: 32732788

[33] Gee KM Jones RE Casson C Barth B Troendle D Beres AL. More is less: the advantages of performing concurrent laparoscopic cholecystectomy and endoscopic retrograde cholangiopancreatography for pediatric Choledocholithiasis. J Laparoendosc Adv Surg Tech A. (2019) 29:1481–5. doi: 10.1089/lap.2019.0429, PMID: 31566486

[34] Rancan A Andreetta M Gaio P Cananzi M Rossoni R La Pergola E. "rendezvous" procedure in children with Cholecysto-Choledocholithiasis. J Laparoendosc Adv Surg Tech A. (2019) 29:1081–4. doi: 10.1089/lap.2018.0696, PMID: 31237499

[35] Overman RE Jr Hsieh LB Thomas TT Gadepalli SK Geiger J. Pediatric laparoscopic common bile duct exploration: an opportunity to decrease ERCP complications. J Surg Res. (2019) 242:318–22. doi: 10.1016/j.jss.2019.04.072, PMID: 31129240

[36] Muller CO Boimond MB Rega A Michelet D El Ghoneimi A Bonnard A. Safety and efficacy of one-stage total laparoscopic treatment of common bile duct stones in children. Surg Endosc. (2015) 29:1831–6. doi: 10.1007/s00464-014-3872-4, PMID: 25318361

[37] Lau BJ Sydorak RM Shaul DB. Laparoscopic techniques for safe and successful removal of common bile duct stones in pediatric patients. J Laparoendosc Adv Surg Tech A. (2014) 24:362–5. doi: 10.1089/lap.2013.0174, PMID: 24195783

[38] Menon S Patel B Saekang E Thomas G Soundappan S Shun A. Laparoscopic exploration of the common bile duct to relieve choledocholithiasis in children. Pediatr Surg Int. (2011) 27:537–40. doi: 10.1007/s00383-010-2826-8, PMID: 21290137

[39] Zaka-ur-Rab A Zaka-ur-Rab Z Fareed R Ahmed I. Cholangitis and choledocholithiasis after repair of duodenal atresia: a case report. Acta Med Iran. (2011) 49:269–74. PMID: 21713741

[40] Rastogi R Rastogi V. Case report: retroperitoneal biliary fluid collections secondary to common bile duct rupture - an unusual complication of choledocholithiasis in a child. Indian J Radiol Imaging. (2008) 18:232–5. doi: 10.4103/0971-3026.41835, PMID: 19774165 PMC2747443

[41] Yanagisawa S Oue T Odashima T Kuda M Tanabe Y Yokomori K. Cholelithiasis and choledocholithiasis associated with anomalous junction of the cystic duct in a child. J Pediatr Surg. (2007) 42:E17–9. doi: 10.1016/j.jpedsurg.2007.07.032, PMID: 17923183

[42] Bonnard A Seguier-Lipszyc E Liguory C Benkerrou M Garel C Malbezin S. Laparoscopic approach as primary treatment of common bile duct stones in children. J Pediatr Surg. (2005) 40:1459–63. doi: 10.1016/j.jpedsurg.2005.05.046, PMID: 16150349

[43] Shah RS Blakely ML Lobe TE. The role of laparoscopy in the management of common bile duct obstruction in children. Surg Endosc. (2001) 15:1353–5. doi: 10.1007/s004640000320, PMID: 11727149

[44] Tanaka Y Kawaguchi C Mizote H Yano H. Biliary tract duplication accompanied by choledocholithiasis: report of a case. Surg Today. (1999) 29:1168–71. doi: 10.1007/BF02482267, PMID: 10552336

