# Supplementary material for: Laparoscope combined with ureteroscope in the treatment of bile duct stones and cholecystolithiasis in a child: a case report and literature review
Source: Front Med (Lausanne). 2024 Oct 3;11:1429127. doi: 10.3389/fmed.2024.1429127 (PMC11483362; doi:10.3389/fmed.2024.1429127)
Supplement: Supplementary file 1 [file Table_1.DOCX]

| **Table S1.** Clinical features and surgical methods of previous cholecystolithiasis with bile duct stones in children | | | | | | | | | | | | | |
| --- | --- | --- | --- | --- | --- | --- | --- | --- | --- | --- | --- | --- | --- |
| **Study** | **Country** | **Age; years** | **Gender-male;(n%)** | **Sample size; n** | **Symptom;(n%)** | **Past medical history;(n%)** | **Diameter of stone; mm** | **Diameter of CBD; mm** | **Total bilirubin; mg/dl** | **Direct bilirubin; mg/dl** | **Alkaline phosphatase; U/L** | **Surgical methods** | **Complication;(n)** |
| Rauh,2024 | America | median(IQR):15.1(12.6,16.4) | 34(21.8%) | 156 | - | - | - | - | - | - | - | LCBDE (38 with power flush;21 with baskets;38 with balloon sphincteroplasty;9 with choledochoscope) | Pancreatitis (1); Conversion to open surgery (1) |
| Niknam,2023 | Iran | 5 | 1(100%) | 1 | Abdominal pain | - | 15 | 10.8 | 1.7 | - | 962 | ERCP+LC | None |
| Pogorelić,2022 | Croatia | mean(range):14(11.5-14.5) | 1(16.7%) | 6 | Abdominal pain (100%); Jaundice (100%) | - | median(IQR):8(6.5,11) | median(IQR):9(8,11) | median(IQR):12.9 (9.1,20.4) | - | median(IQR):1174(1051,1387) | ERCP+LC | Unknown (2) |
|  |  | mean(range):13.5(11-14.5) | 2(14.3%) | 14 | Abdominal pain (92.9%); Jaundice (100%) | - | median(IQR):7.5(6,11) | median(IQR):9(7.5,11) | median(IQR):13.2 (8.9,20.6) | - | median(IQR):1105(1004,1354) | LCBDE | None |
| Cisaro,2022 | Italy | mean(range):10.7(1.67-15) | - | 11 | - | Beta thalassemia (9.1%); Spherocytosis (27.3%); Portal Hypertension (9.1%) | - | - | - | - | - | LC+RV(10);LC+ERCP(1) | Bleeding (1) |
| Fishman,2020 | America | mean(range):15(13-16.5) | 12(18%) | 67 | - | Hemolytic disease (19%) | - | - | mean(range):6(0-46.4) | mean(range):3.6(0-24.9) | - | LC+ERCP | None |
| Gee,2019 | America | mean(range):14.2(3-18) | 8(57.1%) | 14 | - | - | - | mean±SD:7.46±3.2 | mean±SD:5.0±5.4 | mean±SD:2.8±3.9 | - | LC+ERCP | Bleeding (1) |
|  |  | mean(range):14.2(9-17) | 32（76.2%） | 42 | - | - | - | mean±SD:9.0±3.0 | mean±SD:5.0±9.0 | mean±SD:3.4±6.5 | - | LC+ERCP | Bleeding (2); Cholangitis (1); Pancreatic pseudocyst (1) |
| Rancan,2019 | Italy | 11 | 0(0) | 1 | Vomiting; Abdominal pain | Sickle-cell disease | - | 8 | 21 | 15.4 | - | LC+ERCP | Pancreatitis |
|  |  | 10 | 0(0) | 1 | Vomiting; Abdominal pain | Hemolytic disease | 2 | 9 | 15.9 | 9.8 | - | LC+ERCP | None |
| Overman,2019 | America | median:14 | - | 23 | - | - | - | - | - | - | - | LC+ERCP | Bleeding (2); Pancreatitis (2); Readmission (1); Other (1) |
| Muller,2015 | France | mean(range):11(0.25-18) | - | 36 | Abdominal pain (88%); Jaundice (64%) | Hemolytic disease (61%) | - | mean(range):7.5(5-14.5) | - | mean:81 | mean:161 | LCBDE (ureteral catheter is introduced through the catheter in the cystic duct) | Conversion to open surgery (1); ERCP was performed after (6) |
| Lau,2014 | America | mean(range):9.5(0.25-13) | 3(43%) | 7 | - | - | - | - | - | - | - | LCBDE (1 without a scope, 3 with choledochoscope,3 with ureteroscope) | None |
| Short,2013 | America | median:15 | 3(17%) | 18 | Abdominal pain (78%) | - | - | - | median:1.2 | median:0.85 | - | LCBDE (choledochoscope is introduced through the catheter in the cystic duct) | Retained Stone(3)；Pancreatitis (1); Recurrence (1) |
|  |  | median:13.4 | 6(25%) | 24 | Abdominal pain (92%) | - | - | - | median:1.2 | median:0.45 | - | LC+ERCP | Duodenal Perforation (1); Bleeding (1); Retained Stone (2); Pancreatitis (2) |
| Hill,2013 | America | mean(range):14(0.33-20) | - | 52 | - | - | - | - | - | - | - | LCBDE (choledochoscope is introduced through the catheter in the cystic duct) | Conversion to open surgery (4) |
| Menon,2011 | Australia | mean(range):12.5(10-14) | - | 8 | - | Hereditary spherocytosis (12.5%) | - | - | - | - | - | LCBDE (ureteral catheter is introduced through the catheter in the cystic duct) | ERCP/ES was performed after (2) |
| Zaka-ur-Rab,2011 | India | 6 | 1(100%) | 1 | Abdominal pain; Jaundice; Fever | Duodenoduodenostomy for duodenal atresia type | 14 | 17 | 5.5 | 4 | 2870 | LCBDE | None |
| Rastogi,2008 | India | 10 | 1(100%) | 1 | Abdominal pain; Jaundice | - | - | - | elevated | elevated | elevated | LCBDE | None |
| Yanagisawa,2007 | Japan | 10 | 0(0) | 1 | Abdominal pain; Fever | - | 7 | - | 4.83 | 3.81 | 2099 | LC+ERCP | None |
| Bonnard,2005 | France | mean(range):9.9(0.25-15.5) | - | 12 | Jaundice (58.3%) | Nephrotic syndrome (8.3%); Sickle cell disease (25%); G6P deficiency (8.3%); Minkowski-Chauffard (25%); Thalassemia (25%) | - | mean(range):5.2(3-8) | - | - | mean(range):747(528-1044) | LCBDE (ureteral catheter is introduced through the catheter in the cystic duct) | Conversion to open surgery (1); ERCP/ES was performed after (4) |
| Shah,2001 | America | mean(range):11.6(5-16) | - | 5 | - | - | - | - | - | - | - | LCBDE (7-Fr multichannel rigid and 10-Fr flexible fiberoptic cystoscope) | ES was performed after (1) |
| Tanaka,1999 | Japan | 13 | 0(0) | 1 | Abdominal pain | Duplicated common bile duct | 1 | - | - | - | - | LCBDE | None |
| CBD: common bile duct, LCBDE: laparoscopic common bile duct exploration, ERCP: endoscopic retrograde cholangiopancreatography, LC: laparoscopic cholecystectomy, IQR: Inter quartile range, ES: endoscopic sphincterotomy; RV: rendez-vous | | | | | | | | | | | | | |
